# Community Pharmacy Involvement in Cardiovascular Patient Follow-Up: A Conceptual Framework for Integrated Ambulatory Pharmaceutical Care

**DOI:** 10.3390/healthcare14193152

**Published:** 2026-09-23

**Authors:** Lúcia Santos, Cristina Gavina, Jorge Oliveira, Maria João Lima, Edite Teixeira-Lemos

**Affiliations:** 1 Portuguese Pharmaceutical Society, Central Regional Section, Rua Castro Matoso 12A, 3000-104 Coimbra, Portugal; lucia.santos@ordemfarmaceuticos.pt; 2 REQUIMTE/LAQV—Research Unit on Applied Molecular Biosciences, Pharmacy Faculty, University of Coimbra, Azinhaga de Santa Comba, 3000-548 Coimbra, Portugal; 3 Medicine Department, Faculty of Medicine, University of Porto, Alameda Prof. Hernâni Monteiro, 4200-319 Porto, Portugal; cristina.gavina@gmail.com; 4 Cardiology Department, Local Health Unit Matosinhos, 4464-513 Senhora da Hora, Portugal; 5 CERNAS-UPV Research Center, Polytechnic University of Viseu, Avenida José Maria Vale Andrade, 3504-510 Viseu, Portugal; joliveira@esav.ipv.pt (J.O.); mjoaolima@esav.ipv.pt (M.J.L.)

**Keywords:** community pharmacy, cardiovascular disease, pharmaceutical care, ambulatory care, integrated care, continuity of care

## Abstract

**Background/Objectives:** Cardiovascular disease (CVD) remains the leading cause of morbidity and mortality worldwide, requiring coordinated long-term management of cardiovascular risk factors, complex pharmacotherapy, and continuity of care across healthcare settings. Community pharmacists are well-placed to support ambulatory cardiovascular care; however, their contribution often remains insufficiently integrated into cardiovascular care pathways, limiting continuity, information exchange, and coordinated medication management across community, primary, and specialist care. This study aimed to establish the scientific basis for the development of an Integrated Ambulatory Pharmaceutical Follow-Up Programme in Cardiology by synthesising the available evidence on pharmacist-led cardiovascular care. **Methods:** An evidence-informed narrative synthesis was conducted using targeted searches of PubMed/MEDLINE, Scopus, and Web of Science, complemented by manual screening of reference lists. Priority was given to systematic reviews, meta-analyses, randomised controlled trials, and high-quality observational studies addressing pharmacist-led interventions in cardiovascular risk management, heart failure, transitions of care, cardiac rehabilitation, and integrated pharmaceutical care. Evidence was mapped across clinical outcomes, care processes, professional roles, and implementation requirements and subsequently translated into the components of the proposed integrated ambulatory care framework. **Results:** The available evidence consistently indicates that pharmacist-led interventions improve cardiovascular risk factor control, medication adherence, pharmacotherapy optimisation, and patient-reported outcomes. Community pharmacies provide accessible and effective settings for cardiovascular risk screening, chronic disease monitoring, and structured follow-up. In heart failure, pharmacist involvement supports optimisation of guideline-directed medical therapy, while during care transitions it contributes to the identification and reduction in medication-related problems. In cardiac rehabilitation and secondary prevention, pharmacist-led care supports medication optimisation, adherence, and cardiovascular risk-factor management. Overall, the available evidence supports the integration of community pharmacists into multidisciplinary ambulatory cardiovascular care pathways and provides the evidence base for the proposed conceptual framework. **Conclusions:** The proposed Integrated Ambulatory Pharmaceutical Follow-Up Programme in Cardiology provides an evidence-informed conceptual framework to support the structured integration of community pharmacists into ambulatory cardiovascular care. Its implementation has the potential to strengthen continuity of care, optimise pharmacotherapy, and support improved cardiovascular outcomes when supported by collaborative practice models, interoperable health information systems, and appropriate organisational frameworks.

## 1. Introduction

Cardiovascular diseases (CVDs) remain the leading cause of death worldwide, accounting for an estimated 19.8 million deaths in 2022 [1]. Patients with CVD frequently present with multiple modifiable risk factors, including hypertension, dyslipidaemia, diabetes mellitus, and tobacco use, which contribute to disease progression and render long-term management particularly challenging. Adequate control of these risk factors and sustained adherence to guideline-recommended medications are essential to reduce the risk of adverse cardiovascular events; however, real-world adherence to prescribed therapies remains suboptimal [2]. Poor medication adherence contributes to preventable hospitalisation, increased healthcare costs, and diminished quality of life.

Pharmacists are among the most accessible healthcare professionals and frequently serve as the first point of contact for patients with chronic diseases. In community pharmacies, pharmacists are well-placed to provide clinical services beyond medication dispensing, including medication therapy reviews, patient counselling, cardiovascular risk assessment, and long-term monitoring [3]. Over the past two decades, a growing body of evidence has evaluated pharmacist-led interventions across a range of cardiology-related domains. Systematic reviews and clinical studies consistently show that pharmacist-led care improves medication adherence and the management of major cardiovascular risk factors, including blood pressure, lipid levels, and glycaemic control [4,5,6,7,8]. Community pharmacists frequently conduct cardiovascular risk screening programmes and patient education initiatives that promote early detection and risk factor management [9]. Beyond cardiovascular risk-factor control, pharmacist involvement has demonstrated important benefits in more complex areas of ambulatory cardiovascular care. In heart failure (HF), systematic reviews and randomised controlled trials (RCTs) indicate that pharmacist participation supports medication optimisation and adherence to guideline-directed medical therapy (GDMT), improves symptom control, and reduces hospital readmissions [10,11]. Pharmacist-led transitional care services also contribute to medication reconciliation and the identification and management of medication-related problems and adverse drug events following hospital discharge, a period of heightened clinical vulnerability [12,13].

The accumulated evidence highlights the potential of community pharmacists as essential contributors to cardiovascular disease management and to continuity of care across settings. However, despite demonstrated benefits of pharmacist-led interventions, their integration into cardiovascular care pathways remains fragmented, with insufficient coordination between community pharmacies, hospital-based cardiology services, and primary care. This lack of an integrated ambulatory follow-up pathway may limit continuity of care, timely information exchange, coordinated therapeutic decision-making, and longitudinal medication management. This manuscript aims to provide an evidence-informed conceptual foundation for the development and future implementation of an Integrated Ambulatory Pharmaceutical Follow-Up Programme in Cardiology. By linking clinical evidence from multiple cardiovascular domains to health service design, this work seeks to support the structured integration of community pharmacists into ambulatory cardiology pathways to strengthen long-term outcomes, therapeutic adherence, and patient engagement.

## 2. Methods

This manuscript presents an evidence-informed narrative synthesis of the literature undertaken to establish the scientific basis for the development of an Integrated Ambulatory Pharmaceutical Follow-Up Programme in Cardiology. Rather than providing an exhaustive review of the literature, the objective was to identify, appraise, and integrate the most relevant evidence supporting the programme’s principal components, including community pharmacy practice, ambulatory cardiovascular care, medication optimisation, continuity of care, and multidisciplinary collaboration. The methodological approach was structured as a three-stage conceptual framework development process comprising the following: (1) Targeted Literature Identification; (2) Evidence Mapping and Synthesis; and (3) Operational Component Translation.

In the first stage, Targeted Literature Identification, a targeted literature search was performed using major biomedical databases, including PubMed/MEDLINE, Scopus, and Web of Science, supplemented by manual screening of reference lists of key publications. The search primarily encompassed publications from 2008 to 2025, reflecting current models of pharmacist-led cardiovascular care. Only studies published in English were Considered. Searches focused on the core concepts underpinning the proposed framework, including community pharmacy and pharmacist-led care, cardiovascular disease, medication management, continuity and transitions of care, and integrated care. Additional domain-specific terms, including cardiovascular risk-factor management, heart failure, cardiac rehabilitation, medication adherence, pharmacotherapy optimisation, and multidisciplinary care, were used as appropriate. Search terminology was adapted to the indexing and functionality of each database.

Priority was given to systematic reviews, meta-analyses, randomised controlled trials, and relevant observational studies addressing cardiovascular risk factor management, heart failure, care transitions, cardiac rehabilitation, and integrated pharmaceutical care. Study selection and prioritisation were guided by both methodological design and relevance to the evidence domains underpinning the proposed framework. Systematic reviews and meta-analyses were prioritised to capture the broader evidence base, complemented by randomised controlled trials providing direct evidence on specific pharmacist-led interventions. Observational, feasibility, and implementation studies were included when they contributed relevant information on community pharmacy practice, care transitions, service organisation, or implementation that was not adequately captured by higher-level evidence. Observational studies were considered when they included clearly defined study populations, pharmacist-led interventions and relevant clinical or care-process outcomes, appropriate comparators or longitudinal designs where applicable, clearly specified follow-up periods, and analytical methods appropriate to the study design, including adjustment for relevant confounders. Where reported, the completeness of follow-up, handling of missing data, and use of validated outcome measures were also considered when assessing the relevance and methodological quality of the evidence. Studies were retained for the narrative synthesis when they contributed directly to the clinical, professional, organisational, or implementation components of the proposed framework. Only peer-reviewed studies directly relevant to ambulatory cardiovascular care, care transitions, cardiac rehabilitation, or integrated pharmaceutical care were included. Policy reports and guideline documents from international health organisations were also reviewed when pertinent. As the purpose of this work was to develop an evidence-informed conceptual framework rather than to conduct a systematic review or meta-analysis, no formal risk-of-bias assessment or quantitative synthesis of evidence was undertaken. A formal record-by-record systematic screening process and PRISMA-style study flow were therefore not undertaken, and the numbers of records identified, screened, and excluded were not prospectively recorded.

The evidence identified through this process was synthesised narratively in the following sections and used to inform the conceptual design of the proposed Integrated Ambulatory Pharmaceutical Follow-Up Programme in Cardiology.

## 3. Results

### 3.1. Evidence Supporting Pharmacist-Led Cardiovascular Risk Management

The available evidence consistently demonstrates that pharmacist-led interventions improve cardiovascular risk factor management in ambulatory care. Systematic reviews and meta-analyses report significant improvements in medication optimisation, medication adherence, blood pressure control, lipid management, and other clinical outcomes across diverse healthcare settings. For example, Martínez–Mardones et al. [8] conducted a systematic review and meta-analysis of pharmacist-led medication reviews in ambulatory care for patients with cardiovascular risk factors, including hypertension, dyslipidaemia, and diabetes. The review demonstrated significant improvements in cardiovascular risk-factor control, particularly reductions in systolic blood pressure and low-density lipoprotein cholesterol, while highlighting the contribution of pharmacists to medication optimisation, the identification and resolution of medication-related problems, and collaboration with prescribers. These findings reinforce the important role of pharmacists as integral members of multidisciplinary cardiovascular care teams, supporting patient self-management and contributing to more effective long-term cardiovascular risk reduction.

A meta-analysis focusing specifically on community pharmacy interventions demonstrated meaningful reductions in both systolic and diastolic blood pressure, confirming that structured pharmacist-led interventions—including medication reviews, lifestyle counselling, adherence assessment, and regular monitoring—are effective strategies for hypertension management in community settings [4]. Subsequent systematic reviews have reinforced these findings, showing that pharmacist involvement contributes to medication optimisation, improved management of modifiable cardiovascular risk factors, reduced hospital utilisation, and greater adherence to evidence-based therapies [6]. Similarly, pharmacist participation in cardiac rehabilitation and secondary prevention programmes has been associated with improved low-density lipoprotein cholesterol and blood pressure control, enhanced medication adherence, and better patient-reported outcomes [14]. Taken together, these findings consistently demonstrate that pharmacist-led interventions produce clinically meaningful improvements in cardiovascular risk management and support their broader integration into ambulatory cardiology services.

### 3.2. Community Pharmacy Implementation and Operational Feasibility

While the effectiveness of pharmacist-led interventions is now well established, their successful implementation also depends on the healthcare setting in which these services are delivered. Among the available models of care, community pharmacies offer unique advantages for the long-term management of cardiovascular disease because they combine high accessibility with regular patient contact, enabling continuous monitoring, early identification of medication-related problems, and reinforcement of adherence throughout treatment. Community pharmacies are among the most accessible healthcare settings, often serving as the first point of professional contact for individuals with chronic diseases and unmet healthcare needs. Unlike many primary care services, they operate with extended opening hours and generally do not require appointments, allowing patients to access professional healthcare support without barriers such as waiting times or additional costs. This accessibility facilitates frequent interactions between pharmacists and patients, enabling early identification of health problems, ongoing monitoring of therapeutic effectiveness, and continuity of care, all of which are particularly valuable in the long-term management of cardiovascular disease. Evidence further indicates that community pharmacists routinely deliver patient-centred clinical services that extend well beyond dispensing medications.

A recent systematic review of 45 studies found that community pharmacists regularly provide medication therapy monitoring, lifestyle counselling, health education, and cardiovascular risk assessment and management, highlighting their substantial contribution to primary care efforts to prevent and control cardiovascular disease [15]. Across the studies reviewed, pharmacists consistently fulfilled both preventive and clinical management roles, and patients reported high levels of satisfaction with the services provided.

Building on this broader evidence, randomised controlled trials and implementation studies provide more specific evidence that structured cardiovascular care programmes can be integrated into community pharmacy practice. The landmark RxEACH trial, conducted by Tsuyuki et al. [16] (Table 1) across 56 Canadian community pharmacies, showed that pharmacists with expanded clinical authority—including independent prescribing, medication optimisation, and laboratory monitoring—achieved significant improvements in systolic blood pressure, low-density lipoprotein cholesterol, glycaemic control, and smoking cessation, resulting in a meaningful reduction in overall estimated cardiovascular risk. These findings provide robust evidence supporting the integration of community pharmacists into structured ambulatory cardiovascular care.

Subsequent implementation studies have confirmed that community pharmacies can successfully deliver cardiovascular services when supported by collaborative practice agreements, shared clinical documentation, and structured follow-up protocols. For example, a multisite practice transformation programme in the United States demonstrated significant improvements in blood pressure control and medication optimisation, highlighting the feasibility of implementing pharmacist-led cardiovascular services in routine clinical practice [17] (Table 1).

Taken together, these findings support community pharmacy as a feasible setting for structured cardiovascular follow-up, combining accessibility and regular patient contact with medication management, risk-factor monitoring, patient education, and opportunities for collaboration with other healthcare professionals. The principal findings from the studies supporting the clinical effectiveness and implementation of pharmacist-led cardiovascular care are summarised in Table 1.

### 3.3. Heart Failure and Outpatient Cardiology

Pharmacists are increasingly recognised for their clinical contributions in the management of heart failure (HF) and in outpatient cardiology, where the complexity of pharmacotherapy, the need for close monitoring, and the elevated risk of clinical deterioration create significant opportunities for impactful pharmaceutical care. Heart failure remains a leading cause of morbidity, mortality, and unplanned hospitalisation worldwide, requiring rigorous titration of GDMT, intensive patient education, and sustained medication adherence, all areas in which pharmacists have demonstrated strong clinical contributions.

Early evidence from the systematic review by Koshman et al. [10] indicated that pharmacist involvement in outpatient HF care reduced HF-related and all-cause hospitalisations while improving adherence and patient self-management. Over the subsequent decade, larger and more sophisticated studies have continued to validate these findings. A meta-analysis by Schumacher et al. [6] confirmed that pharmacist-led HF interventions consistently improve medication optimisation and facilitate faster, more complete titration of GDMT, a critical determinant of mortality and hospitalisation risk. More recently, Wang et al. [11] demonstrated in a randomised controlled trial that a pharmacist-led HF management model improved medication adherence, symptom burden, quality of life, and titration of renin–angiotensin system inhibitors, beta-blockers, and mineralocorticoid receptor antagonists, resulting in meaningful reductions in HF exacerbations and unplanned healthcare utilisation. Similarly, Zakaraya et al. [20] showed that integrating pharmacists into multidisciplinary HF clinics improved GDMT optimisation, reduced therapeutic inertia, and significantly increased adherence, an essential determinant of long-term outcomes in chronic HF.

The benefits of pharmacist involvement extend beyond heart failure to broader outpatient cardiology settings, including coronary artery disease, atrial fibrillation, hypertension, Dyslipidaemia and post-acute coronary syndrome follow-up. Pharmacists routinely perform medication reconciliation, optimise antithrombotic therapy, manage lipid-lowering treatments, and identify polypharmacy-related risks, all of which contribute to safer and more effective long-term cardiovascular care.

### 3.4. Transitions of Care: Hospital to Community Continuity

The period immediately following hospital discharge represents one of the highest-risk phases in the cardiovascular care continuum, particularly for patients with complex conditions such as heart failure, acute coronary syndromes, or arrhythmia, or those requiring antithrombotic therapy. During this transition, patients often face changes to their medication regimens, difficulty understanding new therapies, and elevated risks of medication discrepancies, non-adherence, and adverse drug events. Evidence consistently indicates that up to half of patients encounter at least one medication-related problem within the first 30 days after discharge, underscoring the vulnerability of this period and the need for structured, multidisciplinary support [21].

Pharmacist-led transitional care interventions have proven particularly effective in addressing these challenges. Evidence from Uitvlugt et al. [13] demonstrated that pharmacist-delivered post-discharge follow-up, encompassing medication reconciliation, targeted counselling, and early identification of drug therapy problems, significantly reduced medication-related issues and improved treatment continuity. Similarly, Bonetti et al. [22] found that systematic pharmacist assessment at 30 days post-discharge identified clinically relevant pharmacotherapy problems and mitigated risks associated with complex cardiovascular medication regimens.

The potential impact of pharmacist involvement during care transitions is especially pronounced in cardiology, where therapeutic regimens often require precise titration, coordination of multiple agents, and adherence to GDMT. Patients discharged following acute decompensated HF may have newly initiated therapies such as angiotensin receptor–neprilysin inhibitors (ARNIs), mineralocorticoid receptor antagonists, or sodium–glucose cotransporter-2 (SGLT-2) inhibitors or may require up-titration of beta-blockers and renin–angiotensin system inhibitors [6]. Patients recovering from acute coronary syndromes typically receive dual antiplatelet therapy, lipid-lowering agents, and anticoagulants, all of which require careful monitoring to maximise benefit while minimising the risk of adverse events [23].

Within an integrated outpatient cardiology programme, community pharmacists are ideally placed to act as continuity-of-care anchors during the hospital-to-community transition. Their accessibility, regular patient contact, and detailed knowledge of chronic medication regimens enable them to identify discrepancies between discharge prescriptions and community pharmacy records, reinforce patients’ understanding of new treatments, monitor for early signs of adverse effects or clinical deterioration, and communicate promptly with prescribing clinicians to resolve emerging issues. Emerging evidence suggests that integrating community pharmacists into transitional care pathways not only reduces medication-related problems but may also lower hospital readmission rates, enhance adherence, and improve patient satisfaction with care [24].

### 3.5. Cardiac Rehabilitation and Secondary Prevention

Cardiac rehabilitation (CR) and secondary prevention constitute essential components of long-term cardiovascular care, encompassing supervised exercise, risk factor management, patient education, psychological support, and medication optimisation. Although the benefits of CR, including reduced mortality, improved functional capacity, and prevention of recurrent cardiovascular events, are well established, participation and sustained adherence remain insufficient worldwide. Variations in follow-up care after programme completion contribute to treatment gaps and therapeutic inertia. As multidisciplinary CR models continue to evolve, pharmacists have become key contributors to both formal rehabilitation efforts and post-rehabilitation maintenance.

A recent systematic review by Ahmed et al. [14] demonstrated that pharmacist involvement in CR programmes leads to significant improvements in medication adherence, patient knowledge, and clinical outcomes, including reductions in LDL C and blood pressure. In these settings, pharmacists typically manage medication reconciliation, adherence support, optimisation of cardioprotective therapies, lifestyle counselling, and tailored patient education. Their role is especially important given the complexity of contemporary cardiovascular pharmacotherapy, which frequently encompasses antithrombotic agents, beta-blockers, ACE inhibitors or ARNIs, statins, ezetimibe, PCSK9 inhibitors, and glucose-lowering drugs with established cardioprotective indications.

Beyond formal CR settings, pharmacists also play a pivotal role in secondary prevention within outpatient environments. Community pharmacists, given their accessibility and regular patient contact, are well positioned to reinforce long-term adherence and support risk factor management after CR completion. Research indicates that pharmacist-led interventions following rehabilitation improve persistence with statins, antihypertensive agents, and antiplatelet therapies, while also addressing behavioural risk factors such as diet, smoking cessation, and physical activity [14,15]. This ongoing support is especially important because the most pronounced reductions in adherence typically occur in the months following completion of formal rehabilitation programmes.

Pharmacists also contribute to the identification of medication-related problems during CR, including adverse drug reactions, drug–drug interactions, and therapeutic duplications. Their involvement in CR teams supports comprehensive medication management, reduces preventable hospitalisations, and improves coordination among cardiology services, primary care, and community pharmacy. As healthcare systems move toward integrated, patient-centred cardiovascular care models, expanding the pharmacist’s role across both supervised CR programmes and long-term secondary prevention offers a scalable approach to improving patient outcomes and sustaining adherence to evidence-based therapies.

### 3.6. Integrated Pharmaceutical Care Models

Integrated pharmaceutical care models have gained prominence over the past decade as healthcare systems increasingly adopt multidisciplinary, patient-centred strategies for managing chronic cardiovascular disease. These models aim to reduce fragmentation between care settings, improve therapeutic continuity, and optimise clinical outcomes through coordinated efforts involving pharmacists, physicians, nurses, and other healthcare professionals. The rationale for integrated care stems from the recognition that cardiovascular management, particularly for conditions such as hypertension, dyslipidaemia, diabetes, and heart failure, requires ongoing therapeutic adjustments, adherence support, and proactive identification of medication-related problems. Pharmacists, with their pharmacotherapy expertise and community accessibility, are well-positioned to contribute across this continuum. Pioneering work in primary care settings has demonstrated the effectiveness of integrating pharmacists into multidisciplinary teams. Studies by Hazen et al. [25] and Geurts et al. [26] show that formal pharmacist integration into interdisciplinary primary care, through clinical medication review and shared pharmaceutical care plans, improves medication therapy management and supports patient-centred care. These models emphasise structured collaboration, shared decision-making, and bidirectional information flow, elements equally relevant to outpatient cardiology.

Recent research has explored digital and systemic innovations that facilitate integrated pharmaceutical care. Vervloet et al. [27] evaluated the TRIAGE tool, an electronic decision-support system used within community pharmacies to identify patients at elevated risk of medication-related problems. Their findings showed that digital alerts linked to dispensing data can effectively trigger clinical interventions, improve monitoring, and support targeted follow-up. Similar digital approaches, including shared electronic health records and secure communication platforms, have demonstrated improvements in coordination between hospital cardiology services, primary care, and community pharmacies [28,29]. Integrated models have also been developed specifically within the cardiovascular domain, notably for hypertension and heart failure. Team-based approaches involving pharmacists in outpatient cardiology and HF clinics are associated with better GDMT titration, reductions in therapeutic inertia, and improved adherence [6,11]. Implementation science frameworks indicate that these models succeed when pharmacists are supported by clear clinical protocols, shared care agreements, and interoperable documentation systems that facilitate real-time communication of clinical decisions [30]. Overall, the literature demonstrates that integrated pharmaceutical care models, whether digital, collaborative, or clinic-based, strengthen the cardiovascular care continuum, reduce medication-related harm, improve chronic disease outcomes, and enhance system efficiency.

### 3.7. Proposed Conceptual Framework for an Integrated Ambulatory Pharmaceutical Follow-Up Programme

The evidence synthesised throughout this manuscript provides the scientific foundation for an Integrated Ambulatory Pharmaceutical Follow-Up Programme in Cardiology, conceived as a structured, collaborative model of care that formally incorporates community pharmacists into the ambulatory cardiovascular care pathway (Table 2). Rather than introducing new clinical interventions, the proposed framework integrates established evidence on pharmacist-led cardiovascular care into a coordinated service model designed to strengthen continuity of care across hospital, primary care, and community settings.

The added value of the proposed framework therefore lies not in the novelty of its individual components, many of which have already been implemented and evaluated, but in their integration within a single longitudinal cardiovascular care pathway. Whereas pharmacist-led models have often addressed specific conditions, interventions, or points in the care pathway, the present framework brings together cardiovascular risk-factor management, heart failure pharmacotherapy, transitions of care, cardiac rehabilitation, and long-term secondary prevention within a common organisational structure. These domains are connected through defined professional roles, critical points of contact, structured communication and referral pathways, shared information requirements, and mechanisms for follow-up and escalation. The framework thus provides an evidence-informed organisational structure for coordinating established pharmacist-led activities across community pharmacy, cardiology, and primary care rather than proposing a new set of clinical interventions.

The proposed pathway begins when an eligible patient enters the programme through a clinically relevant point of contact, such as hospital discharge, referral from cardiology or primary care, initiation or modification of cardiovascular therapy, heart failure or post-cardiac rehabilitation follow-up, or identification of relevant cardiovascular or medication-related needs in the community pharmacy. An initial pharmacist assessment includes medication reconciliation and review, assessment of adherence and medication-related problems, review of relevant cardiovascular risk factors and available clinical parameters, patient education, and identification of potential therapeutic optimisation needs. These findings inform an individualised follow-up plan adapted to the patient’s clinical risk, therapeutic complexity, and specific needs.

Subsequent pharmacist follow-up combines scheduled reassessment with additional event-triggered contacts following clinically relevant events, such as hospital discharge, treatment changes, deterioration in clinical status, suspected adverse drug reactions, or adherence or safety concerns. During follow-up, pharmacists monitor relevant cardiovascular and medication-related parameters, support adherence and self-management, identify and address medication-related problems, and communicate therapeutic recommendations where appropriate. Pharmacotherapy optimisation, including GDMT titration, may be undertaken where permitted by the pharmacist’s professional scope of practice and local regulatory framework. When identified problems require diagnostic assessment or therapeutic decisions beyond the pharmacist’s authorised scope of practice, structured referral or escalation to cardiology or primary care is required.

This pathway depends on bidirectional information exchange across community pharmacy, primary care, and cardiology, supported by shared clinical information where available, documented pharmacist assessments and recommendations, and structured referral and feedback mechanisms. Follow-up frequency is intentionally not prescribed uniformly at this conceptual stage but should be adapted to clinical risk, therapeutic complexity, medication changes, care transitions, and individual patient needs. The framework also incorporates measurable process indicators, including completion of scheduled pharmacist contacts, resolution or referral of medication-related problems, documented therapeutic recommendations and their acceptance where applicable, and completion of referrals and communication across care settings. These operational elements are summarised in Table 2 and illustrated as a patient pathway in Figure 1.

## 4. Discussion

The findings of this evidence-informed narrative synthesis suggest that the principal challenge is no longer whether pharmacists can contribute effectively to cardiovascular care, but rather how this contribution can be integrated into coordinated longitudinal care pathways. Despite substantial evidence supporting pharmacist-led interventions across cardiovascular risk management, pharmacotherapy optimisation, heart failure, transitions of care, cardiac rehabilitation, and secondary prevention, these activities frequently remain fragmented across healthcare settings. Limited integration between community pharmacy, cardiology services, and primary care may restrict continuity of care, timely information exchange, coordinated therapeutic decision-making, and longitudinal medication management.

The proposed framework seeks to address this healthcare-delivery gap by translating established evidence on pharmacist-led cardiovascular care into an integrated ambulatory model that connects community pharmacists with cardiology and primary care teams. Rather than introducing new clinical interventions, it integrates existing pharmacist-led activities into a coordinated pathway to support earlier identification of therapeutic problems, reduce clinical and therapeutic inertia, strengthen longitudinal medication management, and improve continuity during vulnerable care transitions, particularly after hospital discharge. Structured communication, referral, and follow-up mechanisms are therefore central to the framework, enabling pharmacist-led interventions to operate as part of coordinated cardiovascular care rather than as isolated services. Geographical context should also be considered when operationalising the framework. Rural and remote populations may face greater distances to cardiology and other specialised healthcare services and have more limited access to multidisciplinary follow-up than those in urban settings. In such contexts, community pharmacies may provide an important accessible point of continuity between patients and other healthcare providers, although local pharmacy capacity, staffing, access to clinical information, and referral pathways may also vary. Accordingly, implementation should be adapted to local service availability, geographical accessibility, and population needs.

The evidence also reinforces the strategic importance of community pharmacies within integrated care pathways. Their accessibility, frequent patient contact, and established relationships with patients enable pharmacists to provide longitudinal support that complements specialist and primary care. This continuity is particularly relevant for cardiovascular disease, where long-term outcomes depend not only on prescribing evidence-based therapies but also on sustained adherence, timely treatment optimisation, and early identification of medication-related problems. Consequently, community pharmacists should be viewed not merely as dispensers of medicines but as clinical partners who support proactive disease management throughout the patient’s care journey.

The added value of this manuscript lies in integrating evidence traditionally considered in separate clinical contexts into a coherent longitudinal framework for pharmacist-led ambulatory cardiovascular follow-up. As detailed in Section 3.7, its contribution is not the introduction of new pharmacist-led interventions, but rather the organisation of existing interventions within a common care pathway linking community pharmacy, cardiology, and primary care across cardiovascular risk management, heart failure, transitions of care, cardiac rehabilitation, and secondary prevention. Previous reviews have largely focused on individual pharmacist interventions in hypertension, heart failure, medication adherence, transitions of care, or cardiac rehabilitation. By synthesising these complementary areas, the present work proposes a unified conceptual framework for an Integrated Ambulatory Pharmaceutical Follow-Up Programme in Cardiology. This provides a structured approach to coordinating established pharmacist-led activities across hospital, primary care, and community settings, with the aim of strengthening continuity of care and multidisciplinary collaboration.

The strength of the supporting evidence is not uniform across components of the proposed framework. Evidence is strongest for cardiovascular risk-factor management and pharmacotherapy optimisation, with systematic reviews, meta-analyses, and randomised controlled trials demonstrating improvements in blood pressure, lipid and glycaemic control, medication adherence, and medication optimisation. Pharmacist involvement in heart failure management is also supported by systematic and randomised evidence, particularly for optimising guideline-directed medical therapy and adherence. By contrast, community pharmacy-based cardiovascular screening and case finding, some transitional care and cardiac rehabilitation models, and the operational integration of pharmacists across care settings rely more heavily on observational, feasibility, practice-based, and implementation studies. These findings remain relevant to framework development but should be interpreted with greater caution regarding effectiveness and generalisability. The available literature is also heterogeneous in intervention content and intensity, pharmacist scope of practice, patient populations, comparators, follow-up duration, healthcare settings, and outcome measures. Differences between healthcare systems, including professional regulation, access to clinical information, prescribing authority, and models of multidisciplinary collaboration, may influence both intervention effectiveness and transferability. Publication bias cannot be ruled out, particularly given the predominance of studies reporting favourable outcomes. The proposed framework should therefore be interpreted as integrating components supported by varying levels and types of evidence, rather than assuming uniform effectiveness across all domains.

From a health systems perspective, the proposed framework has implications that extend beyond individual patient outcomes. Healthcare systems are increasingly challenged by ageing populations, multimorbidity, and growing demands for long-term management of chronic cardiovascular disease. In this context, community pharmacy-based follow-up represents a potentially scalable approach because it builds upon an existing healthcare infrastructure rather than requiring the development of entirely new care infrastructures. Integrating community pharmacists into structured ambulatory pathways may therefore improve coordination between healthcare sectors, reduce therapeutic inertia, optimise medication management, and contribute to more efficient use of healthcare resources. A major challenge, however, is the limited interoperability that often exists between specialist electronic medical records and community pharmacy dispensing or clinical information systems. Restricted access to current diagnoses, laboratory results, therapeutic changes, and discharge information may result in incomplete medication records, medication discrepancies, communication failures, and delays in treatment optimisation. Conversely, information generated during community pharmacy follow-up may not be readily available to cardiology or primary care teams, limiting its contribution to subsequent clinical decision-making. The proposed framework therefore incorporates structured communication and referral mechanisms, shared documentation of relevant clinical and medication-related information, defined escalation pathways for identified problems, and clear allocation of collaborative responsibilities across community pharmacy, cardiology, and primary care. Achieving these benefits will depend on progressive interoperability across health information systems, supportive collaborative practice frameworks, clearly defined professional responsibilities, and sustainable reimbursement mechanisms that recognise pharmacists’ clinical contributions. The economic implications of implementing the proposed framework also warrant specific consideration. Although some pharmacist-led interventions have been linked to reduced healthcare utilisation, the present narrative synthesis was not designed to systematically assess cost-effectiveness, resource utilisation, or reimbursement models. Therefore, the economic sustainability of the integrated framework cannot be established from the available evidence. Implementation would require resources for pharmacist time, training, clinical documentation, digital infrastructure, communication with other healthcare professionals, and programme monitoring. The extent to which these additional requirements are offset by potential reductions in medication-related problems, avoidable healthcare utilisation, or hospital readmissions remains to be determined. Moreover, reimbursement arrangements for pharmacist-provided clinical services vary substantially across healthcare systems and may influence both implementation and sustainability. Prospective evaluation of the framework should therefore include economic outcomes, such as implementation costs, resource utilisation, cost-effectiveness, and the sustainability of different funding and reimbursement models.

The proposed framework is not intended for a specific national healthcare system, including Portugal, but rather as an adaptable conceptual model for ambulatory cardiovascular care across different healthcare settings. Many healthcare systems are moving towards multidisciplinary, patient-centred models in which responsibility for chronic disease management is shared across professional groups. The framework presented here is consistent with this evolution, positioning community pharmacists as active participants within coordinated cardiovascular care pathways rather than as providers of isolated pharmaceutical services. Its operationalisation will, however, necessarily vary according to national and local healthcare structures, professional regulation, reimbursement arrangements, pharmacists’ scope of practice and prescribing authority, and access to clinical and laboratory information. Accordingly, activities such as medication reconciliation, pharmacotherapy optimisation, and GDMT titration cannot be implemented uniformly across healthcare systems. Where pharmacists do not have prescribing authority, their contribution to therapeutic optimisation may instead involve medication assessment, identification of treatment-related problems, structured communication with the responsible prescriber, and referral or recommendations for therapeutic adjustment. Similarly, where direct access to shared health records is unavailable, implementation will require alternative mechanisms for timely exchange of relevant clinical and medication information. The framework should therefore be regarded as functionally transferable but operationally adaptable, with its specific components implemented according to the regulatory, organisational, digital, and financial context of each healthcare system.

Implementation feasibility represents an important consideration for the proposed framework. Although it builds largely on existing community pharmacy infrastructure and established pharmacist-led activities, its implementation would require adequate professional capacity, protected time for clinical follow-up, appropriate training, access to relevant clinical and laboratory information, interoperable digital systems, and clearly defined communication and referral pathways. Feasibility is also likely to vary according to pharmacists’ scope of practice, local regulatory frameworks, organisational arrangements, and available funding. Potential barriers include increased workload, limited access to clinical information, duplication of activities across professional groups, unclear allocation of responsibilities, and variable engagement of patients and healthcare professionals. If these issues are not adequately addressed, unintended consequences could include fragmented or duplicated care, communication failures, and additional workload without corresponding improvements in continuity or clinical outcomes. Accordingly, the proposed framework should be regarded as a conceptual model requiring prospective feasibility and implementation testing before its effectiveness, scalability, and sustainability can be established.

Several limitations should be acknowledged. The use of an evidence-informed narrative synthesis was an intentional methodological choice aligned with the objective of this work. Rather than estimating the pooled effect of a single intervention, this approach enabled evidence from diverse clinical, organisational, professional, and care-transition contexts to be mapped and integrated, and subsequently translated into a conceptual model for coordinated ambulatory cardiovascular care. The structured approach adopted—including targeted literature identification, predefined evidence domains, explicit eligibility criteria, evidence mapping, and operational translation—was intended to enhance transparency and reproducibility in the framework development process. Nevertheless, as no formal systematic-review methodology or risk-of-bias assessment was undertaken, the selection and interpretation of the literature involved a degree of expert judgement and may therefore be subject to selection bias. Furthermore, because no formal assessment of the certainty of evidence or publication bias was undertaken, distinctions between stronger and more limited areas of evidence in this manuscript reflect the nature and consistency of the available study designs rather than a formal grading process. Second, the proposed framework remains conceptual and has not yet been prospectively evaluated or tested for feasibility in routine clinical practice. Its effectiveness, feasibility, and generalisability will depend on contextual factors such as healthcare organisation, professional regulation, digital infrastructure, and funding mechanisms, which vary considerably across healthcare systems.

Future research should focus on translating this conceptual framework into clinical practice through implementation and effectiveness studies. In addition to evaluating traditional clinical outcomes, future investigations should assess implementation fidelity, patient and professional acceptability, cost-effectiveness, and the impact of digital interoperability on multidisciplinary collaboration. Such research will be essential to determine whether the benefits observed in pharmacist-led interventions can be replicated within an integrated ambulatory cardiovascular follow-up programme and sustained over the long term.

## 5. Conclusions

This manuscript synthesises the available evidence supporting community pharmacist involvement in cardiovascular disease management and translates it into an evidence-informed conceptual framework for an Integrated Ambulatory Pharmaceutical Follow-Up Programmes in Cardiology. The evidence consistently indicates that pharmacist-led interventions improve cardiovascular risk factor control, medication adherence, optimisation of pharmacotherapy, and patient-reported outcomes across the continuum of cardiovascular care. Community pharmacies, through their accessibility and sustained patient contact, provide an appropriate setting for delivering structured ambulatory cardiovascular services and strengthening continuity of care.

The principal contribution of this work lies in integrating evidence from multiple domains of cardiovascular care—including cardiovascular risk management, heart failure, transitions of care, cardiac rehabilitation, and integrated pharmaceutical care—into a coherent framework that supports the structured involvement of community pharmacists in multidisciplinary ambulatory care pathways. Rather than proposing new clinical interventions, the framework provides an evidence-informed foundation for organising and coordinating pharmacist-led follow-up within routine clinical practice. By defining professional roles, communication and referral pathways, information requirements, escalation processes, and interfaces between community pharmacy, cardiology, and primary care, the framework also provides a practical blueprint to support healthcare administrators and policymakers in developing more integrated ambulatory cardiovascular care pathways.

The successful implementation of this model will require collaborative practice frameworks, interoperable health information systems, clearly defined professional roles, and sustainable reimbursement mechanisms that recognise pharmacists’ clinical contributions. Future implementation research should evaluate not only clinical effectiveness but also feasibility, resource requirements, scalability, acceptability, and cost-effectiveness across different healthcare settings. Further evaluation should also assess how the framework can be adapted to local healthcare infrastructure, reimbursement models, professional regulation, and organisational contexts to support sustainable and coordinated cardiovascular care.

## Figures and Tables

**Figure 1:**
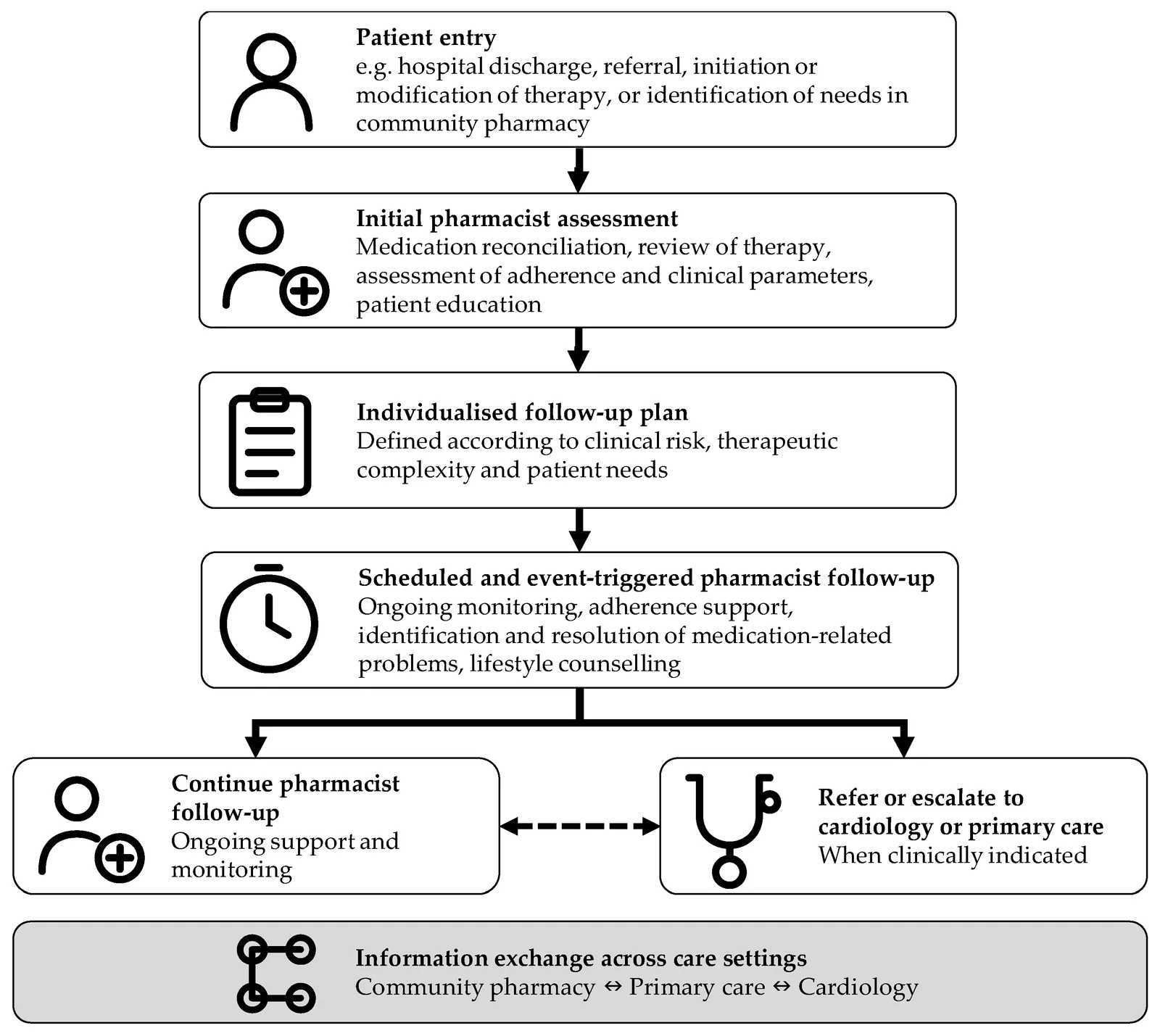
Simplified visual representation of the proposed patient pathway, highlighting the principal pharmacist touchpoints, referral and escalation processes, and bidirectional information exchange across care settings.

**Table 1:** Summary of evidence supporting pharmacist-led cardiovascular risk management and community pharmacy implementation.

Study/ Review	Study Design	Healthcare Setting	Population/Focus	Pharmacist-Led Interventions	Main Findings
[16]	RCT	Community pharmacies (Canada)	High-risk CVD adults	Independent prescribing; medication optimisation; laboratory monitoring; counselling	Significant improvements in SBP, LDL-C, HbA1c, and smoking cessation, resulting in a reduction in estimated overall cardiovascular risk.
[17]	Practice-based intervention	Community pharmacies (USA)	Hypertension and dyslipidaemia	Collaborative agreements; structured protocols; structured follow-up	Improved BP control and increased medication optimisation, supporting the feasibility of structured pharmacist-led cardiovascular care in community pharmacy practice.
[7]	Systematic review (RCTs)	International	CVD populations	Adherence support; counselling; medication review	Improved medication adherence, with favourable trends in BP and LDL-C control.
[15]	Systematic review	Community pharmacies	CVD prevention	Counselling; risk assessment; lifestyle advice	Community pharmacists contributed to cardiovascular risk identification and management, with high levels of patient satisfaction with the services provided.
[18]	Systematic review and meta-analysis of RCTs	General practice and primary care	Adults with hypertension, diabetes and/or dyslipidaemia undergoing primary cardiovascular prevention	Medication review and management; treatment recommendations; patient education; adherence support	Pharmacist-led interventions significantly improved cardiovascular risk-factor control, including SBP, LDL-C, and HbA1c.
[14]	Systematic review	Cardiac rehabilitation and secondary prevention settings	Patients participating in cardiac rehabilitation	Medication optimisation; adherence support; counselling; education; risk-factor management	Pharmacist involvement in cardiac rehabilitation was associated with improvements in clinical and patient-related outcomes, including risk-factor management and medication adherence.
[19]	Cross-sectional study	Community pharmacies, Vienna, Austria	Pharmacy users undergoing cardiovascular risk-factor assessment	Point-of-care testing and assessment of cardiovascular risk factors	Community pharmacy-based assessment identified individuals with previously unrecognised cardiovascular risk factors, supporting the role of community pharmacies in accessible cardiovascular screening and case finding.
[9]	Prospective feasibility study	Portuguese community pharmacies	Adults undergoing cardiovascular risk screening	Cardiovascular risk assessment; screening; referral; patient counselling	Demonstrated the feasibility of cardiovascular risk screening in community pharmacies and identified individuals requiring referral or preventive intervention.
[8]	Systematic review and meta-analysis of RCTs	Community pharmacies and ambulatory care	Adults with hypertension, diabetes and dyslipidaemia	Pharmacist-led medication reviews; medication optimisation; patient follow-up	Pharmacist-led medication reviews improved hypertension, dyslipidaemia, and diabetes control, with significant reductions in SBP and LDL-C, and greater effects observed with advanced medication reviews.

Abbreviations: BP, blood pressure; SBP, systolic blood pressure; LDL-C, low-density lipoprotein cholesterol; HbA1c, glycated haemoglobin; CVD, cardiovascular disease; RCT, randomised controlled trial.

**Table 2:** Conceptual framework for the Integrated Ambulatory Pharmaceutical Follow-Up Programme in Cardiology: core components, implementation requirements, and anticipated outcomes.

Domain	Description
Programme objective	To improve long-term cardiovascular outcomes through structured, pharmacist-led ambulatory follow-up integrated across care settings.
Target population	Adults with established cardiovascular disease or at high cardiovascular risk requiring chronic pharmacotherapy and ongoing clinical follow-up.
Entry points	Hospital discharge; referral from cardiology or primary care; initiation or modification of cardiovascular therapy; heart failure follow-up; post-cardiac rehabilitation follow-up; identification of relevant cardiovascular or medication-related needs in community pharmacy.
Initial pharmacist assessment	Medication reconciliation and review; assessment of adherence and medication-related problems; review of relevant cardiovascular risk factors and available clinical parameters; patient education; identification of therapeutic optimisation needs; establishment of an individualised follow-up plan.
Follow-up contacts	Scheduled reassessment according to clinical risk, therapeutic complexity, medication changes, identified medication-related problems, and patient needs. Additional event-triggered contacts following hospital discharge, clinically relevant treatment changes, deterioration in clinical status, suspected adverse drug reactions, or adherence or safety concerns.
Pharmacist actions	Medication reconciliation and review; adherence assessment and support; cardiovascular risk-factor monitoring; identification and management of medication-related problems; patient education and self-management support; communication of therapeutic recommendations; pharmacotherapy optimisation, including GDMT titration were permitted by professional scope of practice and local regulatory frameworks.
Referral and escalation	Structured referral or communication with cardiology or primary care for clinically significant abnormalities, suspected adverse drug reactions, unresolved medication discrepancies, persistent adherence problems, deterioration in clinical status, or therapeutic initiation, adjustment, or escalation beyond the pharmacist’s authorised scope of practice.
Key actors and care settings	Community pharmacists, cardiologists, primary care physicians, nurses, and other allied healthcare professionals across community pharmacy, outpatient cardiology, primary care, and post-discharge care.
Information exchange	Shared care plans and relevant clinical information where available; documented pharmacist assessments and recommendations; structured referral and feedback pathways; collaborative practice arrangements according to local healthcare structures.
Supporting enablers	Digital health tools; standardised clinical protocols; appropriate access to relevant laboratory and clinical information; professional training and education; supportive regulatory and organisational frameworks.
Process indicators	Completion of scheduled pharmacist follow-up contacts; identification and resolution or referral of medication-related problems; documented recommendations for therapeutic optimisation and their acceptance where applicable; completion of referrals and communication with cardiology or primary care; medication adherence.
Clinical outcomes	Blood pressure and lipid control; heart failure stability; reduced medication-related problems; reduced preventable hospital readmissions.
System-level outcomes	Improved continuity of care; reduced therapeutic inertia; enhanced information exchange and multidisciplinary collaboration across community pharmacy, primary care, and cardiology.

## Data Availability

No new data were created or analysed in this study. Data sharing is not applicable to this article.

## Abbreviations

The following abbreviations are used in this manuscript:

ACE	Angiotensin-converting enzyme
ARNI	Angiotensin receptor–neprilysin inhibitor
BP	Blood pressure
CR	Cardiac rehabilitation
CV	Cardiovascular
CVD	Cardiovascular disease
DBP	Diastolic blood pressure
GDMT	Guideline-directed medical therapy
HbA1c	Glycated haemoglobin
HF	Heart failure
LDL-C	Low-density lipoprotein cholesterol
MRA	Mineralocorticoid receptor antagonist
PCSK9	Proprotein convertase subtilisin/kexin type 9
RCT	Randomised controlled trial
SBP	Systolic blood pressure
SGLT-2	Sodium–glucose cotransporter-2
